# Adaptive Lag Smoother for State Estimation

**DOI:** 10.3390/s22145310

**Published:** 2022-07-15

**Authors:** Shashi Poddar, John L. Crassidis

**Affiliations:** 1 Department of Intelligent Machines & Communication Systems, CSIR—Central Scientific Instruments Organisation, Chandigarh 160030, India; 2 Department of Aerospace and Mechanical Engineering, University at Buffalo, The State University of New York, Buffalo, NY 14260-4400, USA; johnc@buffalo.edu

**Keywords:** adaptive smoother, fixed-lag smoother, Kalman filter, attitude estimation

## Abstract

Fixed-lag smoothing has been used across different disciplines for offline analysis in many applications. With rising computational power and parallel processing architectures, fixed-lag smoothers are increasingly integrated into online processing system with small delays. This delay is directly related to the lag-length used in system design, which needs to be chosen appropriately. In this work, an adaptive approach is devised to choose an appropriate lag-length that provides a good trade-off between accuracy and computational requirements. The analysis shown in this paper for the error dynamics of the fixed-lag smoother over the lags helps in understanding its saturation over increasing lags. In order to provide the empirical results, simulations are carried out over a second-order Newtonian system, single-axis attitude estimation, Van der Pol’s oscillator, and three-axis attitude estimation. The simulation results demonstrate the performance achieved with an adaptive-lag smoother as compared to a fixed-lag smoother with very high lag-length.

## 1. Introduction

State estimation approaches for obtaining estimates from their noisy measurements have been an active research area for decades in applications ranging across control, guidance and communication. Kalman filters have been very popularly used for estimating the state of the multi-dimensional process with an underlying assumption of Gaussian noise. Several filtering approaches have been discussed in the literature towards estimating the true state of a system that is non-linear and added with non-Gaussian distribution of noise as well [1,2,3]. Along with this, some of the research articles have aimed at improved estimation with uncertain system models and noise interference, such as interactive multi-model methods [4] and smooth variable structure filter [5]. However, the ability of these filters cannot substitute for applications that require post-processing of past information to improve accuracy and can only be done by smoothers. Smoothing approaches have been formulated as a specific solution to the Gaussian process and can be divided into essentially three types: fixed-point, fixed-interval or fixed-lag smoothing [6]. Of these three smoothers, fixed-lag smoothing is often preferred over the other two because it can process the incoming data in an online fashion [7]. Fixed-lag smoothing leads to improved accuracy at the cost of a fixed delay between measurements associated with a signal and its estimate [8]. Although early realizations of fixed-lag smoothers were unstable, this issue was later resolved by designing them as a finite-dimensional linear system [9]. Kim proposed finite memory-based fixed-lag smoothers that use only the most recent finite measurements in the window, which is less complex than the traditional fixed-lag smoother [10]. Chen et al. proposed a smooth variable structure smoother that is based on a smooth variable structure filter and aims at overcoming the uncertainties in the system model [11]. Recently, Alenlov and Olsson proposed a design framework by which the particle filter’s lineage degeneracy problem can be solved using a fixed-lag smoother [12]. In a particle filter-based smoother, if the lag-length is small, the forgetting tendency of the model does not kick in, whereas if the lag-length is large, the estimate will lead to high variance. However, they cannot be translated for the case of a simple Kalman filter-based fixed-lag smoother as the one proposed here.

Fixed-lag smoothers have been used for identifying time-varying process models based on input–output observations [13], vehicle side slip angle estimation [14], gravity anomaly estimation [15,16] and pre-processing of geophysical data [17]. The fixed lag smoothers marginalize the old data in the localization pipeline for fusing information from different sensors such as inertial sensors, visual camera [18], ultra-wide band location systems [19], indoor localization using multiple sensors [20], visible light positioning using a photodiode and camera [21] and target tracking in a wireless network environment [22]. The fixed-lag smoothers have also been applied in the case of out of sequence measurements to improve tracking accuracy of incoming targets and increasing the probability of engaging targets [23]. Fixed-lag smoothing has also been popular in the field of tracking a maneuvering target with different motion models such as constant velocity and constant acceleration, and accordingly devise different lag-lengths based on their complexity [24]. Duong et al. applied the smoothing operation for reducing the error built up during the integration of GNSS and camera for motion estimation [25]. The regression problem can also be reformulated as a state estimation problem and solved using smoothers for a specific family of covariance function wherein the computational complexity grows only by O(n) [26,27].

Although several research works have been carried in the area of fixed-lag smoothers, as per the authors’ knowledge, none of them are aimed at finding a suitable lag-length for a given system model. It is hypothesized here to determine a suitable lag-length, beyond which a marginal improvement occurs in the estimation accuracy and is therefore not worth the computational complexity involved in going beyond this specific lag-length. To the best of our knowledge, we provide the first mechanism of adaptively selecting a lag-length for Kalman filter-based smoothers that varies with the system model and noise parameter. It is a simple, yet powerful, fixed-lag smoother modification that helps in achieving confidence in the estimated output. Similar to the filters, smoothers also have initial errors and stochastic errors. Once the transient errors are handled with the initial few lags, only errors due to the noise term remain, resulting in persistent errors. The dynamics of the fixed-lag smoother error is therefore analyzed here to provide a foundation for selecting an appropriate lag value. The task of determining suitable lag-length after which there is only a minimal change in the trace of error covariance is the main novelty of this work. With the help of Monte-Carlo simulation on different system models, the estimation accuracy achieved for a smoother with adaptive lag length is found to work similar to that of smoothers with very high lag length. The remainder of this paper is organized as follows. In Section 2, the fixed-lag smoother equations are defined. Section 3 presents the convergence analysis of several examples, as well as the selection of an appropriate lag value. Finally, Section 5 concludes the paper.

## 2. Fixed-Lag Smoother and Its Dynamic Error

Consider a linear discrete time system given by the following equations:

(1a) $$\begin{array}{c} x_{k}=F_{k-1}x_{k-1}+w_{k} \end{array}$$

(1b) $$\begin{array}{c} z_{k}=H_{k}x_{k}+v_{k} \end{array}$$

Here, $x_{k}$ is the system state at the kth time instant, $F_{k}$ and $H_{k}$ are the state transition matrix and measurement output matrix, respectively, $z_{k}$ is the measurement at the kth time instant, and $w_{k}$ and $v_{k}$ are the process and measurement noise terms, respectively, which are assumed to be zero-mean Gaussian white-noise uncorrelated processes. Here, $Q_{k}=E\left(w_{k}w_{k}^{T}\right)$ is the process noise covariance matrix and $R_{k}=E\left(v_{k}v_{k}^{T}\right)$ is the measurement noise covariance matrix.

The fixed-lag smoother is an optimal smoother that estimates the system state at the (k−N)th time instant given measurements until time *k*, and a fixed lag of *N*. It is therefore desired to obtain the state estimate denoted by ${\hat{x}}_{k-N,k}$ at each time instant, given the measurements, i.e., $z_{1},\;z_{2},\ldots ,\;z_{k}$. This is represented mathematically as ${\hat{x}}_{k-N,k}=E\left(x_{k-N}|z_{1},\;z_{2},\ldots ,\;z_{k}\right)$. The augmented state vector at the kth time instant with a window size *N* includes *N*-lagged states represented as: $X_{k}={\left[x_{k}^{T},\;x_{k,1}^{T},\ldots ,\;x_{k,N+1}^{T}\right]}^{T}$. Here, $x_{k,m}$ is defined as the state $x_{k-m}$ propagated with the identity transition matrix to time *k*. With this definition, the augmented system $X_{k+1}={\left[x_{k+1,0}^{T},\;x_{k+1,1}^{T},\;\ldots ,\;x_{k+1,N+1}^{T}\right]}^{T}$ is defined as

(2a) $$\begin{array}{c} X_{k+1}=\underset{\phi_{k}}{\underset{︸}{\left[\begin{array}{cccc} F_{k} & 0 & \cdots & 0\\ I & 0 & \cdots & 0\\ \vdots & \vdots & \vdots & \vdots \\ 0 & \cdots & I & 0 \end{array}\right]}}X_{k}+\left[\begin{array}{c} I\\ 0\\ \vdots \\ 0 \end{array}\right]w_{k} \end{array}$$

(2b) $$\begin{array}{c} z_{k}=\underset{H_{k}}{\underset{︸}{\left[\begin{array}{cccc} H_{k} & 0 & \cdots & 0 \end{array}\right]}}X_{k}+v_{k} \end{array}$$

The state transition matrix and the sensitivity matrix for the augmented system are represented by $\Phi_{k}$, and $H_{k}$, respectively. These individual states in $X_{k}$ are defined through $X_{k}={\left[x_{k,0}^{T},\;x_{k,1}^{T},\ldots ,\;x_{k,N-1}^{T}\right]}^{T}$ such that the second subscript represents the lag-length.

The Kalman filter estimate for the system is given as

(3) $${\hat{X}}_{k+1}=\Phi_{k}{\hat{X}}_{k}+L_{k}\left(z_{k}-H_{k}{\hat{X}}_{k}\right)$$

Here, ${\hat{X}}_{k+1}={\left[{\hat{x}}_{k+1}^{T},\;{\hat{x}}_{k,k}^{T},\;\ldots ,\;{\hat{x}}_{k-N,k}^{T}\right]}^{T}$ and ${\hat{X}}_{k}={\left[{\hat{x}}_{k}^{T},\;{\hat{x}}_{k-1,k-1}^{T},\ldots ,\;{\hat{x}}_{k-N-1,k-1}^{T}\right]}^{T}$ are the system states at the k+1th and kth time instants, respectively. The individual elements of the augmented system state can therefore be represented in a filtering framework for the jth lag as

(4) $${\hat{x}}_{k-j,k}={\hat{x}}_{k-j-1,k-1}+L_{k,j+1}\left(z_{k}-H_{k}{\hat{x}}_{k}\right)$$

The matrix $L_{k}$ in Equation (3) is the smoother gain matrix with each of its components, i.e., $L_{k,j}$, corresponding to the gain matrix of the individual system states of ${\hat{X}}_{k}$ defined as

(5) $$L_{k}=\left[\begin{array}{c} L_{k,0}\\ L_{k,1}\\ \vdots \\ L_{k,N+1} \end{array}\right]=\left[\begin{array}{c} F_{k}P_{k}^{0,0}H_{k}^{T}\\ P_{k}^{0,0}H_{k}^{T}\\ \vdots \\ P_{k}^{0,N}H_{k}^{T} \end{array}\right]{\left(H_{k}P_{k}^{0,0}H_{k}^{T}+R_{k}\right)}^{-1}.$$

Here, $R_{k}=E\left(v_{k}v_{k}^{T}\right)$ is the measurement noise covariance matrix. It is seen that the estimate of $x_{k+1,N+1}$ is equal to the estimate of $x_{k-N}$ given the measurement up to time *k*. The term $P_{k}$ is the smoother error-covariance matrix which is represented by

(6) $$P_{k}=\left[\begin{array}{ccc} P_{k}^{0,0} & \cdots & {P_{k}^{0,N+1}}^{T}\\ \vdots & \ddots & \vdots \\ P_{k}^{0,N+1} & \cdots & P_{k}^{N+1,N+1} \end{array}\right]$$

with each of its elements computed as

(7) $$P_{k}^{i,j}=E\left[\left(x_{k-i}-{\hat{x}}_{k-i,k-1}\right){\left(x_{k-j}-{\hat{x}}_{k-j,k-1}\right)}^{T}\right]$$

The error-covariance matrix propagation equation for the augmented system can be written as [28]

(8) $$P_{k+1}=\Phi_{k}P_{k}\left(\Phi_{k}-H_{k}^{T}L_{k}^{T}\right)+Q_{k}$$

Substituting the Kalman gain parameters, the diagonal and off-diagonal elements of $P_{k+1}$ for i=1,2,…,N+1 are given by

(9a) $$\begin{array}{c} P_{k+1}^{i,i}=P_{k}^{i-1,i-1}-P_{k}^{0,i-1}H_{k}^{T}L_{k,i}^{T}F_{k}^{T} \end{array}$$

(9b) $$\begin{array}{c} P_{k+1}^{0,i}=P_{k}^{0,i-1}{\left(F_{k}-L_{k,0}H_{k}\right)}^{T} \end{array}$$

Equations (2a)–(9b) describe the fixed-lag smoother for a given system, which provides the estimate ${\hat{x}}_{k,N}$, i.e., the estimate of $x_{k-N}$ given measurements until the kth time instant. For more details on the derivation of the fixed-lag smoother, one can refer to [28].

## 3. Adaptive Lag Selection Mechanism

In Ref. [7], it is stated that a lag-length should be chosen such that it achieves more than 95% performance improvement possible with smoothing. In [29], a theoretical proof is presented for the convergence of a linear Kalman filter in which the filter error dynamics have a fundamental role. The  a posteriori error for a Kalman filter is defined as

(10) $${\tilde{x}}_{k}≜x_{k}-{\hat{x}}_{k}=x_{k}-\left[F_{k-1}{\hat{x}}_{k-1}+K_{k}\left(z_{k}-H_{k}F_{k-1}{\hat{x}}_{k-1}\right)\right]$$

where $K_{k}$ is the Kalman gain matrix. Equation (10) can be rearranged as

(11) $${\tilde{x}}_{k}=\left(I-K_{k}H_{k}\right)\left(x_{k}-F_{k-1}{\hat{x}}_{k-1}\right)-K_{k}v_{k}$$

The dynamic error of the Kalman filter is shown to remain bounded if the initial estimation error, as well as the error due to noise terms, are small [30]. The error for the estimated state at the Nth lag can be represented as

(12) $${\tilde{x}}_{k-N,k}≜x_{k-N}-{\hat{x}}_{k-N,k}$$

Substituting Equation (4) in Equation (12), the error can be rewritten as

(13) $${\tilde{x}}_{k-N,k}=x_{k-N}-\left[{\hat{x}}_{k-N-1,k-1}+L_{k,N+1}\left(z_{k}-H_{k}{\hat{x}}_{k}\right)\right]$$

This is one of the possible representations of the smoother error dynamics. On rearranging, this does not yield a form similar to Equation (11), and hence the dynamics of the smoother error over increasing lags are analyzed using the trace of error-covariance of the smoother directly. Simulations carried out over different problems indicate that during the initial few lags, the smoother attempts to capture information from previous lagged estimates, and once this estimate converges, higher-lag estimates are not very helpful. It is therefore proposed here to adapt the lag-length of the system on the basis of the trace of error-covariance.

The lag-length of fixed-lag smoothers is generally chosen based on available system hardware resources, but not much has been studied towards detection of a reliable lag-length. It is well-known that the trace of the error-covariance matrix is an important parameter to determine a scalar measure of the estimation accuracy. It is also used to indicate the performance of a fixed-lag smoother with a direct relation between the increasing lag and the decreasing trace of the error-covariance matrix. In [7], it is mentioned that a lag-length need not be chosen that achieves more than 95% performance improvement possible with smoothing. It is also suggested to have a lag-length that is 2 to 3 times the time constant of a filter. However, determining the time constant of a dynamic and real-time system is not always plausible. In this work, it is therefore proposed to use the trace of the fixed-lag smoother error-covariance matrix, $\mathrm{tr}(P_{j,k})$, to select an adaptive lag-length value in a heuristic manner. Here, $P_{j,k}=P_{k}^{j,j}$ is the error-covariance of the jth lag at the kth time instant.

The adaptive lag selection approach aims at finding the lag-length at which the $\mathrm{tr}(P_{j,k})$ value saturates and has insignificant changes after a specific lag-length. In order to arrive at a suitable lag-length, the error-covariance ($P_{j,k}$) value at each lag-point is monitored for saturation. The mechanism to detect saturation at any time instant *k* is defined as

(14) $$\left|\mathrm{tr}(P_{j,k})-\mathrm{tr}(P_{j+\alpha ,k})|\leq p\mathrm{tr}(P_{j,k}\right)$$

Here, $P_{j,k}$ is a performance index of smoother with *j* lags at the kth time instant, α a user defined lag constant, and *p* is a scalar parameter that defines the level at which the algorithm should trigger saturation, indicating the percent change occurring with increasing lag. This equation indicates a logical *True* when $P_{j,k}$ saturates and there is not a p% change from the jth to (j+α)th lag. The value of *p* needs to be chosen empirically, as per the variation in $P_{j,k}$ for the specific problem at hand, and largely depends on the noise parameters. For the simulations carried out in this paper, values of α=10 and p=0.005 are selected. The saturation of the error-covariance over lags is monitored only once in a while and repeated after a few instants for dynamic systems. The adaptive lag-length value, denoted AL, determined through this process remains fixed for a system with constant process and measurement noise covariance values, and can be used to determine a suitable lag value as described in Algorithm 1. 
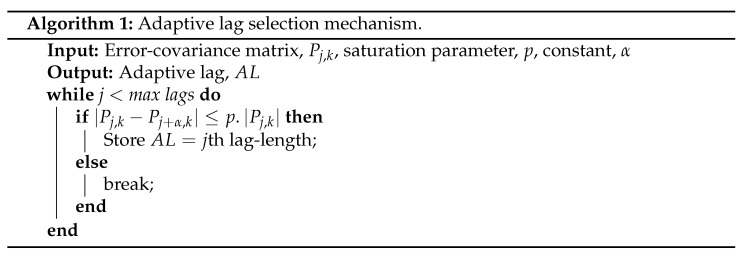


## 4. Simulation Results

The proposed adaptive lag smoother (ALS) scheme of arriving at a suitable lag-length automatically is applied to the problem of: (a) a second-order Newtonian System, (b) single-axis attitude estimation, (c), Van der Pol’s oscillator, and (d) three-axis attitude estimation.

### 4.1. Second-Order Newtonian System

A simple second-order Newtonian system is represented as

(15a) $$x_{k+1}=\left[\begin{array}{cc} 1 & \Delta t\\ 0 & 1 \end{array}\right]x_{k}+\left[\begin{array}{c} \frac{\Delta t^{2}}{2}\\ \Delta t \end{array}\right]\left(u_{k}+w_{k}\right)$$

(15b) $$y_{k}=\left[\begin{array}{cc} 1 & 0 \end{array}\right]x_{k}+v_{k}$$

where Δt is the sampling interval. The system states x(1), position, and x(2), velocity, are commanded by an external acceleration input $u_{k}$ with an acceleration noise $w_{k}$, which is a zero-mean Gaussian white-noise process with standard deviation $\sigma_{w}$. The process-noise, $w_{k}$, is defined as $w_{k}={\left[\frac{\Delta t^{2}}{2}\;\;\;\Delta t\right]}^{T}{\tilde{u}}_{k}$.

(16) $$w_{k}=\left[\begin{array}{c} \frac{\delta t^{2}}{2}\\ \delta t \end{array}\right]{\tilde{u}}_{k}$$

The process-noise covariance, $Q_{k}$, is given by

(17) $$Q_{k}=\left[\begin{array}{cc} \frac{\Delta t^{4}}{4} & \frac{\Delta t^{3}}{2}\\ \frac{\Delta t^{3}}{2} & \Delta t^{2} \end{array}\right]\sigma_{w}^{2}$$

The fixed-lag smoother is applied on this system with Δt=0.1, $\sigma_{w}=2$, measurement noise variance of 4, initial state vector $x_{1}={\left[0\;\;\;0\right]}^{T}$ and initial error-covariance of $I_{2\times 2}$. A total lag-length of N=200 is used to compare the ALS to the fixed-lag smoother with lag=200. The simulation values are adapted from the fixed-lag smoothing problem described in the example 9.1 from [28].

**Figure 1:**
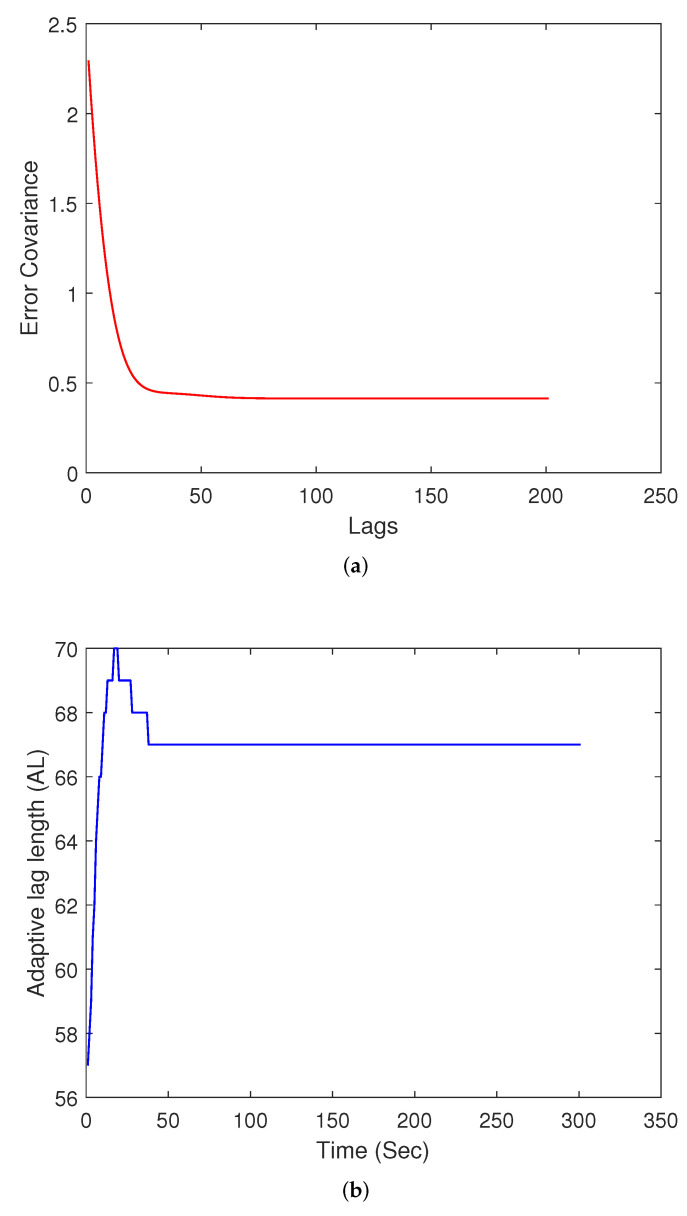
(**a**) Saturation of the error-covariance over increasing lag at k=300, (**b**) Varying adaptive lag-length over time.

The trace of the error-covariance matrix, tr(P) for the fixed-lag-smoother at k=300, is plotted in Figure 1a. It can be seen that the trace of error covariance decreases exponentially for the initial few lags, after which it remains nearly constant. The tr(P) is a performance indicator for the smoother that helps in understanding that the lag-length beyond a specific point will not lead to any significant improvement in the estimation accuracy. In order to arrive at this specific lag point, a saturation detection mechanism as described in Equation (14) is used.

During the initial lags, the AL value varies and then saturates to a value that remains nearly constant, as seen in Figure 1b. Figure 1b plots AL for the case of process noise equal to 2 and measurement noise equal to 4 at different time instants. The performance indicator of the ALS is quantitatively measured by taking the ratio of tr(P) with the highest lag in the simulation, *N*, to that of the tr(P) with the adaptive length parameter, AL, represented as

(18) $$\%\;\mathrm{improvement}=\frac{\mathrm{tr}(P_{N,k})}{\mathrm{tr}(P_{AL,k})}$$

**Figure 2:**
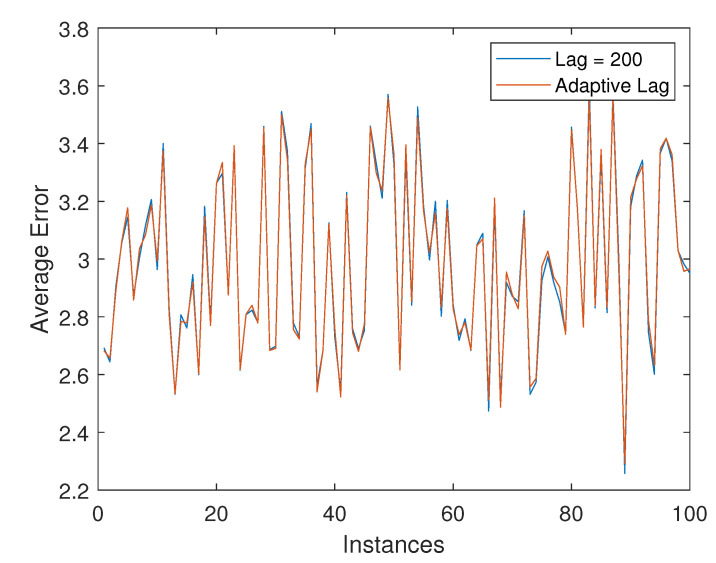
Error comparison between highest lag smoother and adaptive lag smoother.

The performance level achieved through the ALS as compared to a smoother with lag-length equal to 200 for different combinations of acceleration and measurement noise is tabulated in Table 1. The percent improvement metric is the mean of the percent improvement computed at all time instants for a smoother with AL as compared to a smoother with 200 lags. In order to verify the efficacy of the ALS approach, the sum of absolute error between the truth and adaptive lag smoother estimate is compared with the error between the truth and highest lag smoother estimate obtained in the simulation. The error value averaged over the total time is plotted for different instants using a 100 Monte-Carlo run simulation, with results shown in Figure 2. The average absolute error between the highest lag smoother and ALS is nearly the same at different instances, and is a strong indicator of the performance achieved through an ALS as compared to a very high lag smoother.

### 4.2. Single-Axis Attitude Estimation

The single-axis attitude estimation problem involves using attitude angle measurements and gyro rate information, which is simulated to test the validity of the ALS approach. The attitude angle and gyro observations are corrupted with noise and error sources that are compensated using a Kalman filtering approach. The attitude rate, $\dot{\theta }$, is related to the gyro measurement, $\tilde{\omega }$, by

(19) $$\dot{\theta }=\tilde{\omega }-b-\eta_{v}$$

where *b* is the random drift rate and $\eta_{v}$ is a zero-mean Gaussian white-noise process with spectral density given by $\sigma_{v}^{2}$. The random drift rate is modeled as $\dot{b}=\eta_{u}$; where $\eta_{u}$ is a zero-mean Gaussian white-noise process with spectral density $\sigma_{u}^{2}$. Synthetic measurements are created by using a constant angle rate of 0.0011 rad/sec and a sampling interval of 1 s. The noise parameters used in the simulation are $\sigma_{n}=30\times 10^{-5}$ rad, $\sigma_{u}=\sqrt{10}\times 10^{-7}$ rad/s${}^{3/2}$, $\sigma_{v}=\sqrt{10}\times 10^{-4}$ rad/s${}^{1/2}$, and the initial error-covariance matrix is set to $P_{0}=\mathrm{diag}\left(\left[\begin{array}{cc} 1\times 10^{-4} & 1\times 10^{-12} \end{array}\right]\right)$. The simulation set-up is adapted from example 3.3 of [6].

**Figure 3:**
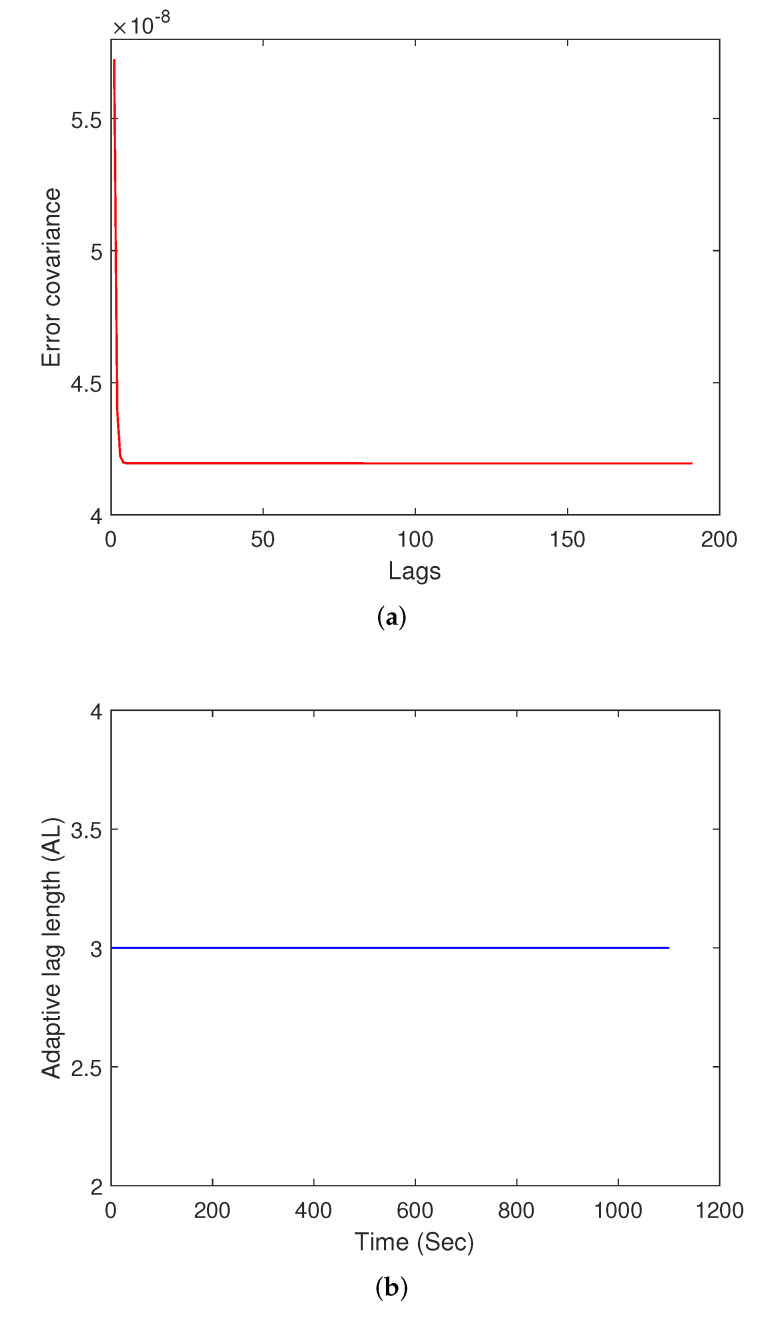
(**a**) Saturation of the error-covariance over increasing lag at k=300, (**b**) varying adaptive lag-length over time for single-axis attitude estimation.

Figure 3a plots the variation for the trace of the error-covariance, tr(P), with increasing lags at the k=300 time instant. The tr(P) parameter saturates after a few lags, thereby indicating that very high lags are not required, and only adds to the computational complexity. Figure 3b plots the value of AL obtained through the adaptive lag selection approach. It is interesting to note here that the ALS settles down with a lag value and remains the same for rest of the simulation period. The performance level achieved for a fixed-lag smoother with lag equal to 200 is compared to that of the ALS by taking the ratio of the tr(P) at specific lag levels for a particular time instant, k=300, and is depicted in Table 2. These simulations indicate that it is worthwhile to stop the smoother at a specific lag point rather than extending it to large lag-lengths and increasing the computational complexity.

### 4.3. Van der Pol Oscillator

Van der Pol’s oscillator equation is a nonlinear differential equation proposed by Van der Pol while experimenting with the oscillations in a vacuum tube. This problem has special characteristics of an oscillating error-covariance over time, unlike other problems that have a monotonically decreasing error-covariance. A generic representation of this nonlinear equation is given as

(20) $$m\ddot{x}+2c\left(x^{2}-1\right)\dot{x}+kx=0$$

where *m* is the mass, *c* is the damping parameter and *k* is the spring parameter. Converting this to a state-space formulation with $x={\left[x\;\;\;\dot{x}\right]}^{T}$ gives

(21a) $$\begin{array}{c} \dot{x_{1}}=x_{2} \end{array}$$

(21b) $$\begin{array}{c} \dot{x_{2}}=-2\left(c/m\right)\left({x_{1}}^{2}-1\right)x_{2}-\left(k/m\right)x_{1} \end{array}$$

The linearized model matrix, *F* and *G* are given by

(22) $$F=\left[\begin{array}{cc} 0 & 1\\ -4\left(c/m\right){\hat{x}}_{1}{\hat{x}}_{2}-\left(k/m\right) & -2\left(c/m\right)\left({{\hat{x}}_{1}}^{2}-1\right) \end{array}\right],\;G=\left[\begin{array}{c} 0\\ 1 \end{array}\right]$$

The measurement output is position only, so H=[10]. Parameters m=c=k=1 are assumed here with an initial condition of $x_{0}={\left[1,\;\;\;0\right]}^{T}$. A sampling interval of 0.01 s and measurement noise standard deviation of 0.01 are used, The synthetic states are generated for 30 s. The model parameters for the extended Kalman filter are assumed to be m=1, c=1.5, and k=1.2, introducing an error in the system model, which is overcome by tuning the process-noise covariance matrix. This process-noise covariance matrix is set to be Q=diag([00.2]), and the initial error-covariance is set to $P_{0}=100I_{2\times 2}$.

**Figure 4:**
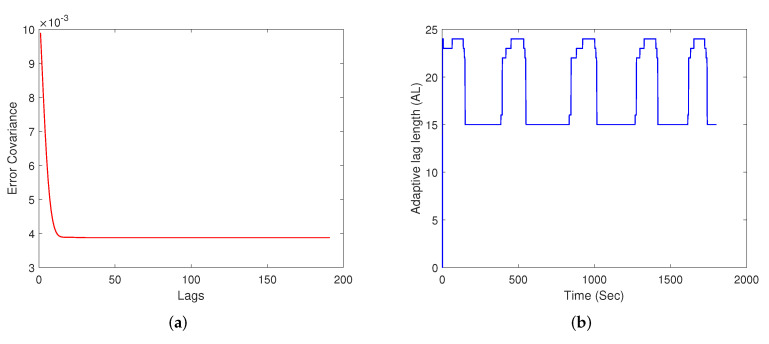
Adaptive lag smoother analysis for Van der Pol Oscillator. (**a**) Varying trace of the error-covariance with increasing lag at k=300. (**b**) Varying adaptive lag-length over time.

A plot of the varying trace of the error-covariance value over increasing lags at k=300 is shown in Figure 4a. The trace of error-covariance value saturates after a few lags and remains constant thereafter, indicating no major change once a specific lag has reached. However, the AL value keeps changing with time in a cyclic fashion and does not remain constant unlike other cases. This is due to the time-varying error-covariance of the nonlinear Van der Pol system over time, and is most likely due to the observability of the associated nonlinear nature of this problem. Table 3 compares the performance of the ALS approach with that of the highest lag smoother N=200 for different combination of process and measurement noise. In this case, the AL value keeps varying between minimum and maximum lag-lengths, and it is recommended here to choose the maximum lag-length for the complete duration for better performance.

The experimentation carried out here proves the efficacy of ALS as compared to very high lag-length fixed lag smoothers.

### 4.4. Three-Axis Attitude Estimation

The ALS approach is now tested on the three-axis attitude estimation problem. This problem is nonlinear in nature, which provides a realistic example. The attitude matrix, *A*, maps from the reference frame to the vehicle body frame according to Ar, where r is a component vector given with respect to the reference frame. The attitude is subsequently parameterized by the quaternion q. The quaternion is a four-dimensional vector defined as $q={\left[\varrho^{T}\;\;\;q_{4}\right]}^{T}$, defined as

(23) $$q\equiv \left[\begin{array}{c} \varrho \\ q_{4} \end{array}\right]$$

with $\varrho \equiv {\left[q_{1}\;\;\;q_{2}\;\;\;q_{3}\right]}^{T}=e\sin\left(\vartheta /2\right)$ and $q_{4}=\cos\left(\vartheta /2\right)$,

(24a) $$\begin{array}{c} \varrho \equiv {\left[q_{1}\;\;\;q_{2}\;\;\;q_{3}\right]}^{T}=e\sin\left(\vartheta /2\right) \end{array}$$

(24b) $$\begin{array}{c} q_{4}=\cos\left(\vartheta /2\right) \end{array}$$

where **e** is the unit Euler axis and ϑ is the rotation angle [31]. A quaternion parameterizing an attitude satisfies a single constraint given by ∥q∥=1. In terms of the quaternion, its associated attitude matrix is given by

(25) $$A\left(q\right)=\left(q_{4}^{2}-{\left||\varrho |\right|}^{2}\right)I_{3\times 3}+2\varrho \;\varrho^{T}-2q_{4}\left[\varrho \times \right]$$

where $I_{3\times 3}$ is a 3×3 identity matrix. The matrix [ϱ×] is the standard cross-product matrix with

(26) $$\left[\varrho \times \right]\equiv \left[\begin{array}{ccc} 0 & -q_{3} & q_{2}\\ q_{3} & 0 & -q_{1}\\ -q_{2} & q_{1} & 0 \end{array}\right]$$

With the attitude parameterized by the quaternion **q**, the physical model is then the quaternion kinematics, given by

(27) $$\dot{q}=\frac{1}{2}\Xi \left(q\right)\omega =\frac{1}{2}\Omega \left(\omega \right)q$$

where ω is the angular rate vector and

(28a) $$\begin{array}{c} \Xi \left(q\right)\equiv \left[\begin{array}{c} q_{4}I_{3\times 3}+\left[\varrho \times \right]\\ -\varrho^{T} \end{array}\right] \end{array}$$

(28b) $$\begin{array}{c} \Omega \left(\omega \right)\equiv \left[\begin{array}{ccc} -[\omega \times ] & & \omega \\ -\omega^{T} & & 0 \end{array}\right] \end{array}$$

(29) $$\Xi \left(q\right)\equiv \left[\begin{array}{c} q_{4}I_{3\times 3}+\left[\varrho \times \right]\\ -\varrho^{T} \end{array}\right]$$

Attitude estimation typically consists of combining the physical model with sensor measurements in order to calculate an attitude trajectory that is, in some sense, stochastically optimal. In addition to attitude sensing hardware, a three-axis gyro is employed to obtain angular rate information. The gyro output $\tilde{\omega }$ is governed by

(30a) $$\begin{array}{c} \tilde{\omega }=\omega +b+\eta_{v} \end{array}$$

(30b) $$\begin{array}{c} \dot{b}=\eta_{u} \end{array}$$

where the vector **b** is the gyro drift, and the vectors $\eta_{v}$ and $\eta_{u}$ are assumed to be zero-mean, Gaussian white-noise processes with spectral densities given by $\sigma_{v}^{2}I_{3\times 3}$ and $\sigma_{u}^{2}I_{3\times 3}$, respectively. The covariance of $w\equiv {\left[\eta_{v}^{T}\;\;\;\eta_{u}^{T}\right]}^{T}$

(31) $$w\equiv \left[\begin{array}{c} \eta_{v}\\ \eta_{u} \end{array}\right]$$

is given by

(32) $$E\{w\left(t\right)w^{T}\left(\tau \right)\}=Q\left(t\right)\delta \left(t-\tau \right),$$

where the spectral density Q(t) is then given by

(33) $$Q\left(t\right)=\left[\begin{array}{cc} \sigma_{v}^{2}I_{3\times 3} & 0_{3\times 3}\\ 0_{3\times 3} & \sigma_{u}^{2}I_{3\times 3} \end{array}\right]$$

where $0_{3\times 3}$ denotes a 3×3 matrix of zeros. The three-axial attitude estimation problem is based on using the quaternion kinematics model, which is represented as:

(34) $$\dot{q}=\frac{1}{2}\Omega \left(\omega \right)q$$

A multiplicative quaternion approach is used to preform the quaternion linearization. The error-quaternion, denoted by $\delta q=[\delta \rho^{T}\;\;\;\delta q_{4}]$, is obtain using $\delta q=q⊗\hat{q}$, where $\hat{q}$ is the estimated quaternion, and ⊗ denotes quaternion multiplication. Considering the multiplication quaternion correction, the linearized form of the derivative of error-quaternion is given by

(35a) $$\begin{array}{c} \delta \dot{\rho }=-\left[\hat{\omega }\times \right]\delta \rho +\frac{1}{2}\Delta \omega \end{array}$$

(35b) $$\begin{array}{c} {\dot{\delta q}}_{4}=0 \end{array}$$

Here, $\delta \dot{\rho }$ and $\delta {\dot{q}}_{4}$ are the derivative of the vector and scalar component of the quaternion, respectively. Since the derivative of the scalar error-portion is zero, then only the vector needs to be considered to develop the EKF, which leads to the multiplicative EKF (MEKF) [32,33]. Substituting $\Delta \omega =\omega -\hat{\omega }=-\left(\Delta b+\eta_{v}\right)$, where $\Delta b=b-\hat{b}$, into Equation (35a) yields a simplified form of linear equations that can be used in EKF:

(36) $$\delta \dot{\alpha }=-\left[\hat{\omega }\times \right]\delta \alpha -\left(\Delta b+\eta_{v}\right)$$

where δα=2δρ is the vector of the first-order roll, pitch and yaw angle errors. This is equivalent to the EKF error model $\Delta {\dot{x}}_{k}=F_{k}\Delta x_{k}+w_{k}$ where $\Delta x_{k}={\left[\delta \alpha_{k}\;\;\;\Delta b_{k}\right]}^{T}$ and

(37) $$Q_{k}=\left[\begin{array}{ccc} \left(\sigma_{v}^{2}\Delta t+\frac{1}{3}\sigma_{u}^{2}\Delta t^{3}\right)I_{3\times 3}\; & \; & -\left(\frac{1}{2}\sigma_{u}^{2}\Delta t^{2}\right)I_{3\times 3}\\ -\left(\frac{1}{2}\sigma_{u}^{2}\Delta t^{2}\right)I_{3\times 3}\; & \; & \left(\sigma_{u}^{2}\Delta t\right)I_{3\times 3} \end{array}\right]$$

Measurements are assumed to be the quaternion with noise. More details of the MEKF can be found in [34].

**Figure 5:**
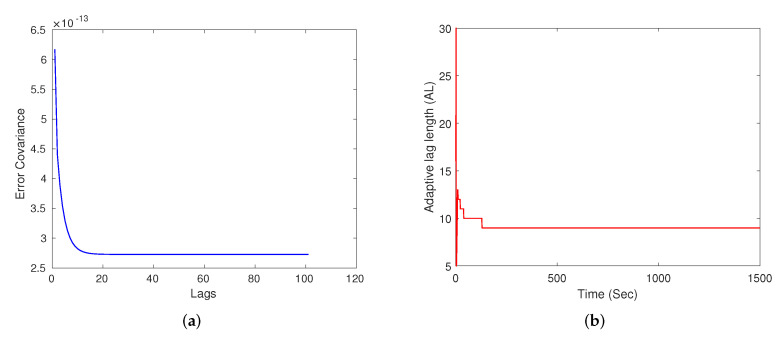
Parameter saturation and varying adaptive lag-length. (**a**) Saturation of parameter value over increasing lag at k=300. (**b**) Varying adaptive lag-length over time.

In order to generate the true attitude, an object is considered to be rotating about its *y*-axis, and the attitude angles are corrupted by the measurement noise, each with a variance of $\sigma_{n}^{2}=8.7266\times 10^{-7}$ rad${}^{2}$. The noise parameters used for the gyro measurements are $\sigma_{u}=\sqrt{10}\times 10^{-10}$ rad/s${}^{3/2}$ and $\sigma_{v}=\sqrt{10}\times 10^{-7}$ rad/s${}^{1/2}$. The initial covariance for the attitude error is set to $P_{0}^{a}=0.1^{2}I_{3\times 3}$ deg${}^{2}=3.0462\times 10^{-6}I_{3\times 3}$ rad${}^{2}$, and the initial covariance for the drift is set to $P_{0}^{b}=0.2^{2}I_{3\times 3}$ (deg/h)${}^{2}=9.4018\times 10^{-13}I_{3\times 3}$ (rad/h)${}^{2}$. These values are taken from example 7.1 in [6]. The output of the adaptive lag smoother is compared with that of the output of the smoother with the highest lag considered in this simulation, which is 100. As seen in Table 4, the output of the adaptive lag smoother is very similar to that of the highest lag smoother, and achieves a performance greater than 99% in most of the cases. The smoother saturates at a specific lag-length, after which not much change occurs in the error-covariance. A plot of varying adaptive lag-length, AL, over time is shown in Figure 5b. The adaptive lag-length varies during the initial time instant, after which it is generally found to settle down to a specific lag-length.

It is also important here to mention that a plot of $E_{c}ov$ over the lag is essential to judge the lag-length and tune the value of *p* accordingly. This *p* parameter is an indicator of percent change that has occurred in the parameter as the lag progresses further. It has also been found that there are cases when the error-covariance changes very gradually with increasing lag-length. Therefore, *a priori* simulations are required for adapting the *p* value accordingly. The simulation shown here shows the efficacy of the ALS as compared to the fixed-lag smoothers with large lags.

## 5. Conclusions

In this work, an adaptive lag smoother approach is proposed that aims at achieving accuracy similar to that of the highest lag smoother with the rationale of small yet appropriate selection of lag-length. It is hypothesized here that once the suitable lag-length is obtained, any further increase of smoothing lag will not lead to any significant improvement in estimator accuracy. The adaptive lag smoother scheme has been tested on position estimation, Van der Pol’s oscillator, and the attitude estimation problem in detail. A convincing performance is achieved with this adaptive scheme, thereby providing a viable solution for determining the appropriate lag-length in practice. The approach also gives a very powerful tool to help design fixed-lag smoothers in which the system model can vary over time. The adaptive lag-length can be determined by running a separate subroutine and provides the maximum number of lags required to achieve convincing performance characteristics.

## Figures and Tables

**Table 1:** Performance comparison of ALS with different noise levels for position estimation system.

Acc. Noise	Meas. Noise	% Improvement	AL
2	4	90.32	67
10	4	99.06	34
2	10	92.03	99
10	10	99.37	50

**Table 2:** Performance comparison of ALS for single-axis attitude estimation with different noise levels.

$\sigma_{n}$ (rad)	$\sigma_{u}$ (rad/s${}^{3/2}$)	$\sigma_{v}$ (rad/s${}^{1/2}$)	% Improvement	AL
$3\times 10{}^{-5}$	$\sqrt{10}\times 10^{-8}$	$\sqrt{10}\times 10^{-5}$	99.87	3
$3\times 10^{-5}$	$\sqrt{10}\times 10^{-9}$	$\sqrt{10}\times 10^{-6}$	99.39	25
$30\times 10^{-5}$	$\sqrt{10}\times 10^{-8}$	$\sqrt{10}\times 10^{-5}$	99.51	25
$30\times 10^{-5}$	$\sqrt{10}\times 10^{-7}$	$\sqrt{10}\times 10^{-4}$	99.91	3

**Table 3:** Performance comparison of ALS for Van der Pol simulation.

Proc. Noise	Meas. Noise	% Improvement	AL
0.2	0.01	99.52	[15–24]
0.05	0.01	99.41	[20–34]
0.2	0.05	98.33	[29–35]
0.05	0.05	98.78	[38–53]

**Table 4:** Performance comparison of ALS with different noise levels for three-axis attitude estimation system.

$\sigma_{n}$ (rad)	$\sigma_{u}$ (rad/s${}^{3/2}$)	$\sigma_{v}$ (rad/s${}^{1/2}$)	% Improvement	AL
$8.73\times 10^{-7}$	$\sqrt{10}\times 10^{-10}$	$\sqrt{10}\times 10^{-7}$	99.33	6
$8.73\times 10^{-7}$	$\sqrt{10}\times 10^{-9}$	$\sqrt{10}\times 10^{-6}$	99.12	7
$8.73\times 10^{-6}$	$\sqrt{10}\times 10^{-10}$	$\sqrt{10}\times 10^{-7}$	99.33	25
$8.73\times 10^{-6}$	$\sqrt{10}\times 10^{-9}$	$\sqrt{10}\times 10^{-6}$	99.12	7

## Data Availability

Not Applicable.

## Nomenclature

$x_{k}$	System state at kth time instant
$F_{k}$	State transition matrix at kth time instant
$z_{k}$	Measurement at kth time instant
$H_{k}$	Measurement output matrix at kth time instant
$w_{k}$	Process noise at kth time instant
$v_{k}$	Measurement noise at kth time instant
$L_{k}$	Smoother gain matrix at kth time instant
$P_{k}$	Error covariance matrix for smoother at kth
$Q_{k}$	Process noise covariance matrix at kth time instant
$R_{k}$	Measurement noise covariance matrix at kth time instant
$K_{k}$	Kalman gain matrix at kth time instant
$P_{k}^{j}$	Error covariance of jth lag at tth time instant
AL	Adaptive lag
**q**	Attitude parameterized by quaternion
$\tilde{\omega }$	Gyroscope measurement corrupted by bias, *b* and noise
